# A machine learning approach to detect potentially harmful and protective suicide-related content in broadcast media

**DOI:** 10.1371/journal.pone.0300917

**Published:** 2024-05-14

**Authors:** Hannah Metzler, Hubert Baginski, David Garcia, Thomas Niederkrotenthaler

**Affiliations:** 1 Section for Science of Complex Systems, Center for Medical Data Science, Medical University of Vienna, Vienna, Austria; 2 Unit Public Mental Health Research, Department of Social and Preventive Medicine, Center for Public Health, Medical University of Vienna, Vienna, Austria; 3 Complexity Science Hub, Vienna, Austria; 4 Institute for Globally Distributed Open Research and Education, Austria; 5 Department of Politics and Public Administration, University of Konstanz, Konstanz, Germany; 6 Institute of Interactive Systems and Data Science, Department of Computer Science and Biomedical Engineering, Graz University of Technology, Graz, Austria; 7 Institute of Information Systems Engineering, Vienna University of Technology, Vienna, Austria; 8 Wiener Werkstaette for Suicide Research, Vienna, Austria; Kitami Institute of Technology, JAPAN

## Abstract

Suicide-related media content has preventive or harmful effects depending on the specific content. Proactive media screening for suicide prevention is hampered by the scarcity of machine learning approaches to detect specific characteristics in news reports. This study applied machine learning to label large quantities of broadcast (TV and radio) media data according to media recommendations reporting suicide. We manually labeled 2519 English transcripts from 44 broadcast sources in Oregon and Washington, USA, published between April 2019 and March 2020. We conducted a content analysis of media reports regarding content characteristics. We trained a benchmark of machine learning models including a majority classifier, approaches based on word frequency (TF-IDF with a linear SVM) and a deep learning model (BERT). We applied these models to a selection of more simple (e.g., focus on a suicide death), and subsequently to putatively more complex tasks (e.g., determining the main focus of a text from 14 categories). Tf-idf with SVM and BERT were clearly better than the naive majority classifier for all characteristics. In a test dataset not used during model training, F1-scores (i.e., the harmonic mean of precision and recall) ranged from 0.90 for celebrity suicide down to 0.58 for the identification of the main focus of the media item. Model performance depended strongly on the number of training samples available, and much less on assumed difficulty of the classification task. This study demonstrates that machine learning models can achieve very satisfactory results for classifying suicide-related broadcast media content, including multi-class characteristics, as long as enough training samples are available. The developed models enable future large-scale screening and investigations of broadcast media.

## Introduction

Current estimates suggest that approximately 700,000 individuals die by suicide each year, making suicide a priority in mental health research and prevention [1]. There are a variety of prevention approaches that have been widely used and recommended, with media reporting about suicide being one of the focal areas in most suicide prevention programmes and strategies [2, 3]. Research suggests that media can influence suicidal thoughts and feelings and potentially, suicidal behaviours in vulnerable individuals, in both a harmful and beneficial direction. For example, a recent-meta-analysis shows evidence for increases of suicides after news about celebrity deaths by suicide within two months after the death, making the reporting of celebrity suicide a core characteristic considered to carry some risk of harmful effects [4]. Specifically, after the reporting of celebrity suicides, suicide rates tend to increase by 13% in one to two subsequent months [4]. Imitation of suicidal behavior such as after celebrity suicide news is commonly referred to as the Werther effect [5]. Studies suggest that other types of media content may have a protective effect. With regard to positive media effects, the strongest evidence exists for stories of hope and recovery from psychosocial crises and suicidal thoughts [6–9]. A recent meta-analysis of randomized controlled trials suggests that media stories that feature individuals who managed to cope with their adversity and suicidal thoughts lead to a small-sized reduction in suicidal thoughts [8]. This beneficial media effect has been coined the Papageno effect and has received increasing attention in prevention and research over the last decade [6].

In order to mitigate the risks associated with suicide-related reporting in broadcast and online media, media recommendations for the reporting on suicide have been developed by national and international public health organizations [3]. These recommendations list content characteristics that have been deemed as potentially harmful and protective. On the one hand, recommendations include suggestions such as not focusing on suicide death; but rather on suicidal ideation in prevention-reporting. Further, they discourage extensive reporting on celebrity death from suicide, and recommend not to perpetuate common public misconceptions and myths about suicide. On the other hand, media recommendations also encourage reporting characteristics that might be helpful to vulnerable audiences. Specifically, they encourage the reporting of alternatives to suicidal behaviour, reporting of healing stories (i.e., stories of individuals who managed to recover from suicidal thoughts), and the reporting of positive outcome of crisis. Although not all of the specific recommendations have solid empirical evidence (many of them are based on expert consensus), current media recommendations are considered to be the most available and practical tool to help prevent suicide from suicide-related media reporting [3].

In order to prevent harm from media reporting on suicide, screening for proactive identification of media reporting has been identified as a priority in suicide prevention [2, 3]. In case of reporting that might do harm, media screening can speed up the process of identifying media reports and potentially distributing media reaching out to media professionals. Such screening, however, has been hampered by the considerable resources needed for content analytic work. Machine learning approaches to automatically detect specific media reporting characteristics might be helpful in this regard. Further, automatic detection of reporting characteristics across a large body of media items would also help with building the evidence base for specific media recommendations, as this would increase the feasibility of testing of how specific media reporting characteristics are associated with suicide and other important outcomes, such as help-seeking.

With regard to broadcast media content, machine learning approaches to categorize content are largely missing. This is in spite of the fact that particularly news media reporting has frequently been found to be associated with suicide [5], and although global efforts to reduce imitation suicide using media recommendations for safe portrayals have largely focused on news media [3]. In contrast, some research has been done on social media content. Specifically, there are now three machine learning studies that categorized Twitter posts into frequent content types, including celebrity suicide posts, posts signaling suicidal ident, awareness campaigns, prevention information, condolences and flippant remarks [10, 11]. These studies have typically used word frequency statistics as predefined features for model training. However, the meaning of words also depends on their context, and more novel deep learning models [see e.g., 11, 12] are needed to take context better into account.

For the present research, we build on previous studies by using a comprehensive human annotation scheme that systematically identifies selected putatively harmful and protective characteristics in broadcast reports about suicide. We selected a number of reporting characteristics from media recommendations for suicide, starting with a difficulty level that we assumed to be more simple (e.g., identifying a report on a celebrity suicide), to increasingly difficult classification tasks (e.g., identifying stories of healing) and ultimately, highly complex tasks such as the identification of the main focus of a news item. Using this selection of content characteristics with different difficulty levels for classification, we want to assess the feasibility of current machine learning approaches for the detection of suicide-related media reporting characteristics.

## Materials and methods

### Media stories transcripts data

We retrieved a total of 2519 transcripts of media items for Oregon and Washington for April 1 2019 to March 30 2020 from the media screening company Infomart/Brandwatch. Only items with a "major focus" on suicide / suicidal ideation / suicide prevention, defined as more than just a short paragraph about the topic, were included. Other items were excluded by Infomart, similar to the approach used in previous related research [13]. The focus on the geographical regions of Oregon and Washington was taken because this study built up on a media evaluation project that included specifically media items from these two states [14]. We reused the transcripts from this previous project to develop the machine learning approach in this study. The dataset was derived from 44 broadcast sources across both states (see [14] and S1 Table for details about sources covered). We received full transcripts of broadcast media items in pdf format.

### Content analysis of media stories

We developed a coding system to assess the quality of media items. The content analysis built on previous analytic approaches for print and online media conducted by members of the study team and was based on the most current versions of media recommendations for the reporting of suicide [3, 6, 13].

The characteristics included reporting elements that are considered to be harmful, as well as characteristics deemed to be relevant for suicide prevention. The process is decribed in detail in Niederkrotenthaler et al., 2022 [14].

#### Intercoder-reliability

Authors ZL, and BT independently coded a random selection of items for each characteristic. Their intercoder-reliability for each characteristic was assessed in terms of percent agreement and Cohen Kappa coefficients. By the end of the process, all codes had substantial agreement (minimum percent agreement: 85%; minimum Kappa: 0.62) with most codes clearly outperforming these minimums. Mean percent agreement was 92.4%; mean Kappa: 0.87. The code book with code definitions and coding examples is provided in S2 Table [14].

#### Content characteristics selected for classification tasks

We developed different machine learning models with the goal of automatically classifying transcripts according to selected reporting characteristics. We selected a pre-defined number of characteristics for the machine learning exercise based on considerations about the difficulty of tasks, starting with characteristics that we assumed to be relatively straight-forward to classify, and proceeding to more complex characteristics. To be selected, characteristics further had to

1. reach sufficient intercoder-reliability in human coding (all characteristics satisfied this criterion; see S2 Table);
2. include characteristics deemed harmful as well as characteristics deemed preventive;
3. ideally, be supported by some evidence for an association with suicides in previous studies.

*Level 1*. We assumed simple binary classification tasks that distinguish between two classes to be easiest, because they likely only depend on detection of specific keywords. A characteristic is either present (positive instances) or not (negative instances). We hypothesized that predicting such characteristics would be simple. We included the following characteristics:

- **Suicide death:** The text includes information on one or several suicide death(s) as one of its focus areas. Stories about suicide death have sometimes been found to be positively associated with suicides [5].
- **Celebrity suicide:** The text reports on suicidal behaviour of a celebrity. The strongest evidence for harmful media effects on suicide comes from studies on the reporting of celebrity suicides [4].

*Level 2*. We assumed this to be binary classification tasks that distinguish between two classes that most likely depend on a rather diverse set of keywords, because different types of keywords may qualify an observation as a positive instance. This often applies for meta-categorical items, which code if one specific example for a type of content is mentioned, e.g., if the text mentions one of multiple possible prevention actions or warning signs. The class depends on what is present, but multiple different contents qualify as a positive instance. We selected the following characteristics for this category:

- **Alternatives to suicide:** The text reports on alternatives to suicidal behaviour. This might include a specific action taken by an individual instead of suicidal behaviour, or a suggestion or advice to seek help [9].
- **Monocausality:** The text gives a monocausal explanation for suicidal behaviour, that is, an explanation relying on exactly one possible motive, cause, or trigger.
- **Positive outcome:** The item reports on a person experiencing a suicide attempt or suicidal ideation, and mastering his/her crisis, or showing or accepting life-affirming or -saving behaviour. The ending is positive [6–9].

There has not been any conclusive evidence about how these reporting characteristics are associated with suicide. Not all of them have been found to be associated with suicides in previous analyses investigating associations of specific media content characteristics and suicide.[6, 13–15]. Nevertheless, such as the suggestion to highlight alternatives for readers and viewers who might be suicidal and to avoid portraying oversimplified reasons for suicide frequently receive specific consideration in media training and have therefore been included in this analysis [3].

*Level 3*. Recent research has increasingly focused on the narrative of specific media stories rather than specific content characteristics. It has been hypothesized that the overall narrative might be more relevant to media effects than specific individual reporting characteristics [16, 17]. Binary characteristics that require the detection of a narrative or the emotional connotation and meaning of a text may be more difficult than level 2 tasks, because the classification depends on how a topic is described in a text. We assumed the following characteristics to require this type of classification:

- **Suicidal ideation:** The text includes information about suicidal ideation as one of its focus areas. Suicidal ideation is the most serious type of suicidal experience mentioned, it is not accompanied by suicide or suicide attempts. Previous studies have found that stories about suicidal ideation in the absence of suicidal behaviours were associated with small decreases in suicides subsequently [6].
- **Healing story:** The narrative is about a healing story, i.e., the process of coping with adversity, hope, and recovery from suicidal thoughts and feelings [8, 9].

There are several randomized controlled trials that indicate that healing stories and positive outcomes of suicidal crises have been found to be associated with reductions in suicidal ideation among vulnerable individuals [9].

- **Myths:** The text enhances a common public myth about suicide (explicitly mentions or implicitly hints at one of nine defined myths). Enhancing means confirming/mentioning without denying it, if a myth is mentioned but debunked, this does not qualify [6].

One study has identified a positive association of portraying public misconceptions related to suicide with subsequent suicides [6].

*Level 4*. Classifying which of multiple classes a text focuses on requires distinguishing between multiple classes, and additionally depends on the extent to which a certain topic is discussed within a text in comparison to other topics (as opposed to the simple presence or absence of a topic). We assumed that two characteristics would be among the most difficult:

- **Problem vs. solution narrative (4 classes):** If the text focuses mainly on suicidal behavior or ideation as a problem, or shows how to find solutions and tackle the problem. If both aspects are described, we compared the emphasis on each in terms of the quantity of devoted text, but also investigated the prominence/framing of both. If they were balanced, we coded both; if neither was mentioned, neither.^18^
- **Main focus of the text (14 classes):** either assisted suicide; suicide prevention advocacy; attempted suicide; suicide cluster; suicide death; healing story; suicidal ideation; legal issues related to suicide; mass murder suicide; murder suicide; policy; prevention; research; other [14].

One study suggests that the framing of suicide as a problem versus solution might have a different effect on the audience. Although this study did not reveal any significant findings for suicide-related outcomes, it identified that individuals reading problem-focused items were more likely to think about concepts related to death and dying as compared to individuals reading prevention-focused media items.^18^ Regarding the main focus of the media item, some focus areas, most importantly healing stories, have been found to be associated with decreases in suicides subsequently [6]. Stories with a main focus on suicide death might be associated with increases in suicides [5]. The detection of the overarching main focus is of high relevance to suicide prevention research and is a complex task based on the sheer number of frequent main content categories. Note that the detection of the main focus is different from other aspects listed above in that the main focus is about the most relevant focal areas of all areas tackled in the text. For example, presence of a healing story is one of the twelve categories but also listed above as a separate characteristic under level 3. In the “main focus” section, only healing stores that make the most important overall focus of the entire item would qualify. Any inclusion of a healing story, no matter which prominence it has within the specific media item, would qualify for “healing story” in the level 3 task.

### Machine learning

We used Python 3.6 for our analysis, including the packages ktrain wrapper [18] for the deep learning library Tensor Flow Keras [19], and the sklearn library [20] for TF-IDF & SVM (see below). For links to the model, the code and data, see the data and code availability statement.

#### Text preprocessing

We apply standard preprocessing strategies. see e.g., [21]. We converted the PDF files into text files using the Linux package pdftotext (https://pdftotext.com/). The original transcripts contained metadata about the radio/TV channel, the time of broadcast, a unique identifier (ID), and the transcribed broadcast. We first separated these metadata from the text using Python, and focused on analysis of the broadcasted text only for all following analyses. Input for BERT (one of the models we trained) is limited to a sequence length of 512 tokens, with remaining tokens being cut off, resulting in information loss. We compensated for this loss by training the word-frequency based models (see below) on the full sequences and comparing their performances to BERT. The majority of the 2519 transcripts in the final sample (83.2%) were shorter than 512 tokens, with a mean length of 316, the 95^th^ percentile at 944, and the 99^th^ percentile at 1411 tokens.

#### Machine learning models and model training

The primary objective of machine learning is to make correct predictions on previously unseen data. To achieve this goal, the dataset is split into three distinct subsets: the training set, the validation set, and the test set. The training set is used for fitting the parameters of the model, the validation set to tune its hyperparameters and for evaluation of the model throughout development. The test set, finally, consists of data that the model has no access to during training, and is used to evaluate the model’s ability to generalize to new data. We divided the dataset of 2519 transcripts into a random training (64%), validation (16%), and test set (20%). Sklearn’s train_test_split was used for this purpose.

We used different text representations and models to classify transcripts:

#### Majority classifier

This is a naive classifier that always predicts the most frequent class.

#### TF-IDF & SVM

Term-frequency-inverse document frequency (TF-IDF) represents text by assigning a weight to each word in a document (transcript) based on how often it appears in this document and how uncommon it is across all documents in a corpus. We used a small modification to the standard formula for TF-IDF by adding 1 in both the numerator and denominator. This change guarantees that every word appears at least once and prevents division by zero [22]:

tf‐idf(t,d)=tf(t,d)*log((N+1)/(df+1))

This value increases proportionally as a word becomes more frequent in a document, and is balanced by the number of documents in the corpus that include the word. This gives more weight to uncommon words, as they are more useful in distinguishing one document from another than words that appear in every document. Using the TF-IDF representation as features, we then trained a linear Support Vector Machine (SVM) classifier. To identify the best combination of parameters, we ran a grid search across different variations of the TF-IDF representation and the SVM classifier. For TF-IDF, these included (1) inclusion of unigrams and/or bigrams, the reduction of text to the top n features by term frequency (10.000, 25.000, 50.000). Inclusion of both uni- and bigrams, and fewer top features (10.000 or 25.000) were better in most classification tasks (see S3 Table for details). Regarding SVM hyperparameters, we tested (1) the applied penalty L_2_,(2) different regularization strength C ∈ (0, 1], (3) automatic adjusting of class weights inversely proportional to their frequency (balanced vs. unbalanced class weights), and (4) the type of kernel (linear, radial) and (5) decision function shapes (one-vs-one and one-vs-rest). Across classification tasks, balanced class weights, a linear kernel, and a one-vs-one decision function shape produced optimal results. The optimal values for regularization strength varied (range: [0.46; 0.91]) between classification tasks (see S3 Table).

#### BERT-base

We used the pre-trained BERT (Bidirectional Encoder Representations from Transformers) base-uncased-model [23], and fine-tuned a separate model for each classification task (i.e., characteristic). BERT is a deep learning language model of the transformer architecture by Google AI. It has 12 transformer layers, 12 self-attention heads, and a hidden size of 768. Its pre-training included a masked language modeling task: a randomly chosen 15% of words in a sentence are masked, and the model learns to predict them from the sequences of words before and after the mask. Additionally, BERT was pre-trained to predict if a sentence follows a given sentence.

We fine-tuned the BERT-base-uncased model by adding a dense output layer that reduces the dimensions of the last layer to the number of labels per classification task, training all parameters simultaneously [24]. The different layers of BERT can capture different levels of semantic and syntactic information, with lower layers probably containing more general information. Therefore, we fine-tuned the layers with different decaying learning rates, following the approach of Howard and Ruder (2018) [25]. To find the maximal learning rate that is associated with a still-falling loss, that is, residuals (prior to the loss diverging), we ran a hyperparameter search. S1 Fig shows that learning rates up to 10e-5 are still associated with falling losses. We report results for BERT with a LR = 1e-5 and text length of 512, for the epoch with best results on the validation set out of maximally 12 epochs (this was usually around epoch 4–7).

#### Evaluation metrics

We indicate precision, recall, and their harmonic mean (the F_1_ score) for each classification task, all of which range from 0 to 1, with 1 indicating high performance. Precision is calculated by dividing the predictions for a specific category (for example, all transcripts labeled as mentioning a characteristic, such as suicide death) by all predictions (for example, those mentioning plus those not mentioning suicide death). It thus indicates how often the model’s label for this specific class is correct.


$$\mathrm{Precision}=\frac{\mathrm{True}\;\mathrm{positives}}{\mathrm{True}\;\mathrm{positives}+\mathrm{False}\;\mathrm{positives}}$$

Recall reflects how many out of all true cases (e.g., transcripts mentioning a suicide death according to human labels) are detected by the model (e.g., labeled as mentioning a suicide death), and corresponds to the sensitivity of a medical test.


$$\mathrm{Recall}=\frac{\mathrm{True}\;\mathrm{positives}}{\mathrm{True}\;\mathrm{positives}+\mathrm{False}\;\mathrm{negatives}}$$

The F_1_-score summarizes precision and recall as the harmonic mean.


$$F_{1}=2\;*\frac{\mathrm{Precision}*\mathrm{Recall}}{\mathrm{Precision}+\mathrm{Recall}}$$

Precision, recall, and F_1_-score are first calculated for each class of a variable (intraclass scores) and can then be averaged across classes to get mean performance scores per classification task (macro-averages).

We report macro-averages for all scores and all models in the validation and test sets. For intraclass performance scores, we provide scores for the best two models, TF-IDF and BERT. We do not report accuracy scores, because our dataset contains mostly characteristics with large imbalances in the sample size between different classes. In such cases, accuracy can be very high even if a classifier always predicts the majority class, and is therefore a biased estimate.

### Ethics statement

The study was conducted in accordance with the Declaration of Helsinki. Because only published public media reports were used for this analysis, review from an Institutional Review Board was not required.

## Results

### Percentage and number of transcripts per category

**Fig 1:**
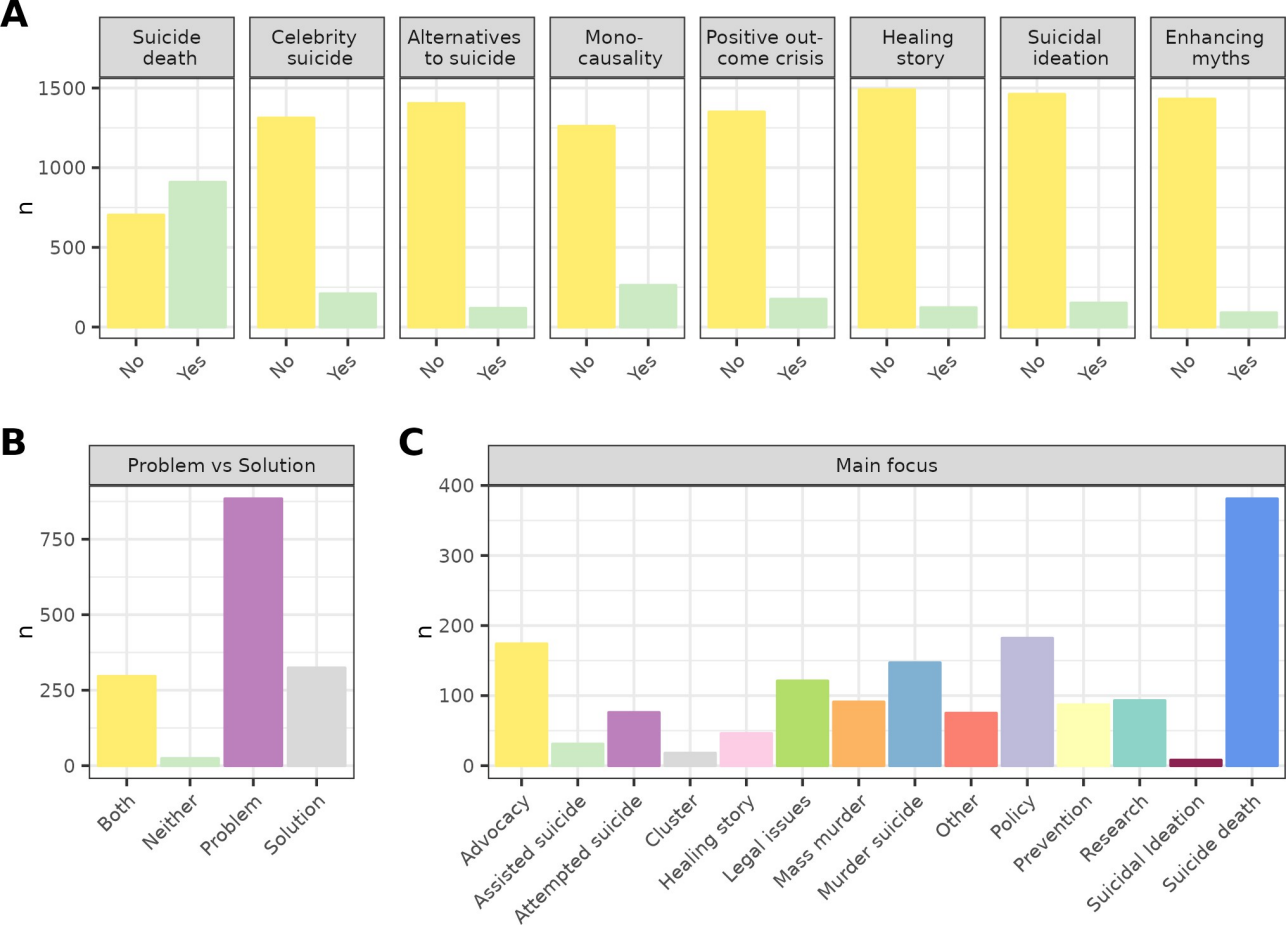
Number of transcripts per category in each classification task in the training set. (A) Binary characteristics. (B) Problem vs. solution narrative. (C) Main focus of a transcript. Proportions were similar across the training (n = 1811), validation (n = 453), and test set (n = 566). S4 Table reports precise sample sizes for the validation and test set. The maximum of y-scales of panel A, B and C differs.

Fig 1 shows the proportion of transcripts per manually coded category for all characteristics, on average across the entire dataset. S4 Table reports the absolute frequency and proportion of transcripts per category separately for the training, validation, and test datasets. Since most texts only contain a few characteristics, the positive instance (the characteristic is present in the media item) is usually a minority class for all binary classification tasks (Fig 1A). Positive instances made up 6 to 14% of all transcripts for binary tasks (class size median = 51.0, min = 28, max = 284 in the test set), with suicide death mentions being the only exception (56.4%, n = 909 training set, n = 284 test set). For the type of narrative (Fig 1B), 57.9% (n = 884 training set, n = 277 test set) of transcripts described suicide predominantly as a problem, whereas 21.2% (n = 324 training set, n = 101 test set) focused on solutions, and 19.4% (n = 296 training set, n = 93 test set) emphasized both aspects equally.

Regarding the main focus of transcripts (Fig 1C), items focusing on suicide deaths were clearly the most frequent (25%). Advocacy (11%), murder-suicide (9.6%), and policy (11.9%) were also among the more frequent focus areas, while healing stories (3%), or suicidal thoughts (0.5%), were rarely the major focus. Given these low percentages, considering the absolute number of examples that models were trained on is crucial before interpreting performance scores. The median number and range of broadcast items for a particular main focus was 89 (min 8, max 381) in the training set, and 27.5 (min 2, max 121) in the test set. The most rarely mentioned main focus areas definitely have too few transcripts in the test set to allow a conclusion about their performance scores. These include assisted suicide (n = 10), suicide clusters (n = 7), healing stories (n = 14), and suicidal ideation (n = 2). Another group of main foci (attempted suicide, mass murder, prevention, research) had below 30 items in the test set, and should be interpreted only with great caution. Only performance for the most frequent main foci, including advocacy (n = 55), suicide death (121), legal issues (n = 38), murder suicide (n = 46), and policy (n = 57) will be reported in the text below. Still, caution is warranted in their interpretation.

### Model comparison

**Fig 2:**
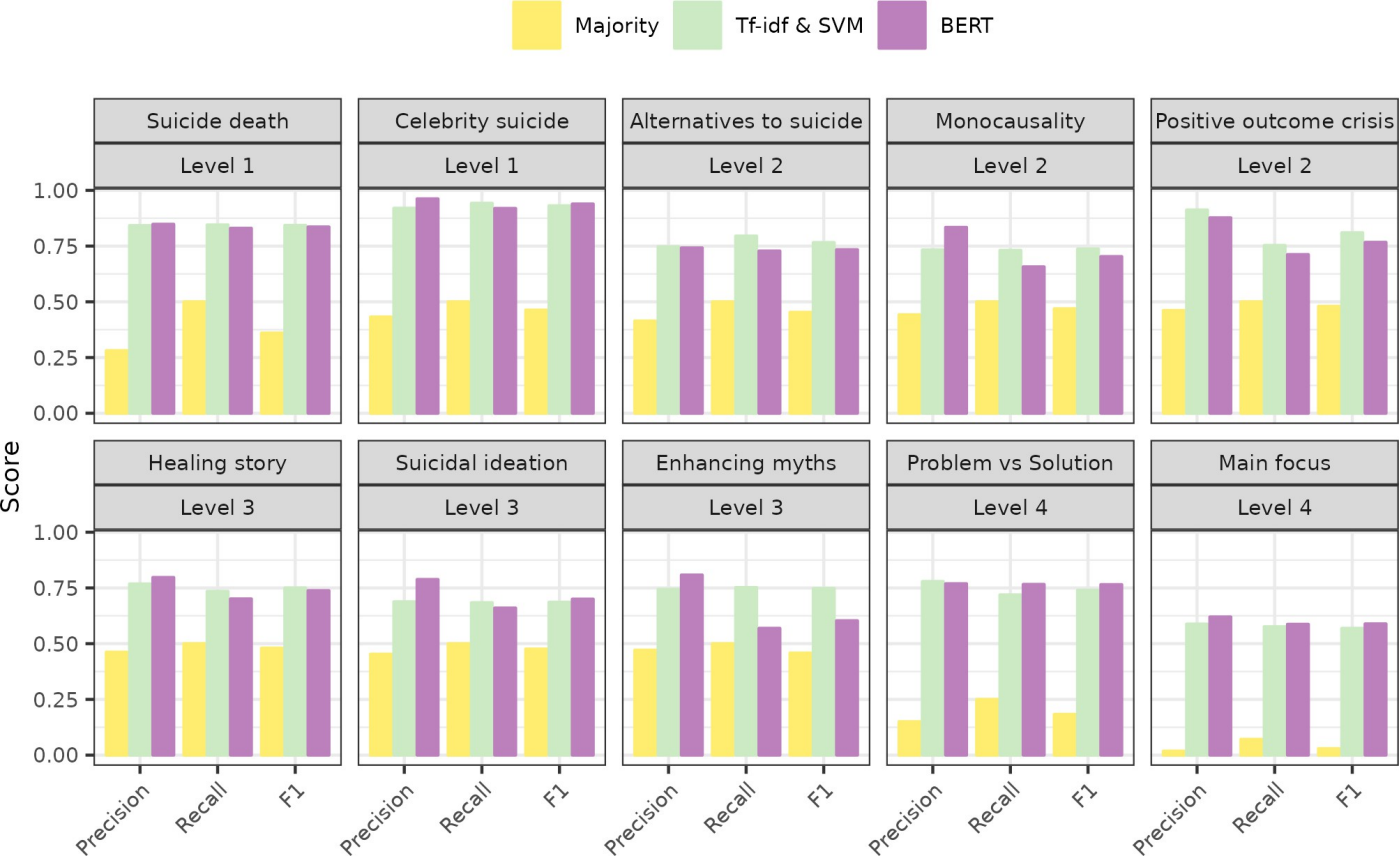
Macro-averages of performance scores (precision, recall, F_1_) in the test set.

Fig 2 illustrates that both Tf-idf with SVM and BERT clearly outperformed the naive majority classifier in all classification tasks. In the following, we therefore focus on BERT and Tf-idf with SVM performances. Fig 2 shows that their performances do not differ substantially for all classification tasks and metrics. In general (but not always), BERT reached higher precision, whereas Tf-idf with SVM reached higher recall for level 2 and 3 classification tasks. As F_1_-scores are a balance of these two, they are overall rather similar for BERT and Tf-idf with SVM.

### Generalization to new data

Across all tasks, performance was comparable across the test and validation set, indicating that both models generalized well to new data (see S5 Table and S1 Fig). For most classification tasks, scores in the two datasets were very similar. When generalizing to the test set, Tf-idf with SVM performance was lowest in the most complex task, determining the main focus of a transcript, achieving .60 instead of .70 for all metrics. BERT’s performance for main focus, although lower in the validation set, remained stable when generalizing to new data in the test set, showing that it learnt generalizable features even when training on multiple categories with few training samples for each. Interestingly, the precision of both models was also by .1 points lower in the validation set for healing stories, but recall was less affected (especially for BERT). This may indicate that healing stories in the training set were not yet representative of the different ways in which stories of healing and coping are described in broadcast media. All further performance scores (recall, precision, and F_1_) below are from the test set, which indicates model performance in new data not used during model training.

### Difficulty of different classification tasks

We assumed classification tasks at level 1 would depend on detection of a few keywords, and thus be easiest. Indeed, Tf-idf with SVM and BERT reached classification performances above .80 for these tasks, and even above .90 for celebrity suicide. Performance scores for level 2 tasks, which we assumed to rely on detection of a larger and more varied set of keywords, were generally around .75, with some better exceptions. For instance, positive outcomes of a suicidal crisis were classified with very high precision (.90) by both models, and monocausal explanations were classified with high precision by BERT (.83). Tasks with level 3 difficulty, which we presumed to rely on the detection of emotional connotation and meaning, were not all clearly more difficult than level 2 tasks for the models. Especially healing stories were as well recognized as other level 2 features by both models (around 0.75 for all balanced metrics). Performance for texts that enhance myths about suicide was similarly good with Tf-idf with SVM (F_1_ = .75), whereas BERT’s performance suffered from low recall (.57). Detecting suicidal ideation, which we had assumed to be a level 3 difficulty task, turned out to be more difficult (F_1_ = .70) than determining the problem vs. solution narrative in a transcript (an assumed level 4 task). Despite having four classes and requiring a comparison between the emphasis on problem vs. solution, Tf-idf’s with SVM F_1_ was .74 and BERT’s F_1_ was .77.

In sum, to reflect actual rather than assumed difficulty levels, some tasks from level 2, 3 and 4 could be reassigned to difficulty levels as follows, based on at least one model’s F1 score lying within the respective range:

- Level 1 (F_1_ [.90, .95]): celebrity suicide
- Level 2 (F_1_ [.80, .85]: suicide deaths, positive outcomes of a suicidal crisis (Tf-idf with SVM)
- Level 3 (F_1_ [.73, .77]: alternatives to suicide, problem vs. solution, healing stories, enhancing myths (Tf-idf), monocausality (Tf-idf with SVM)
- Level 4 (F_1_ ~ .70): suicidal ideation
- Level 5 (F_1_ ~ .58): main focus

### Intra-class performance and correlation with class sample size

**Fig 3:**
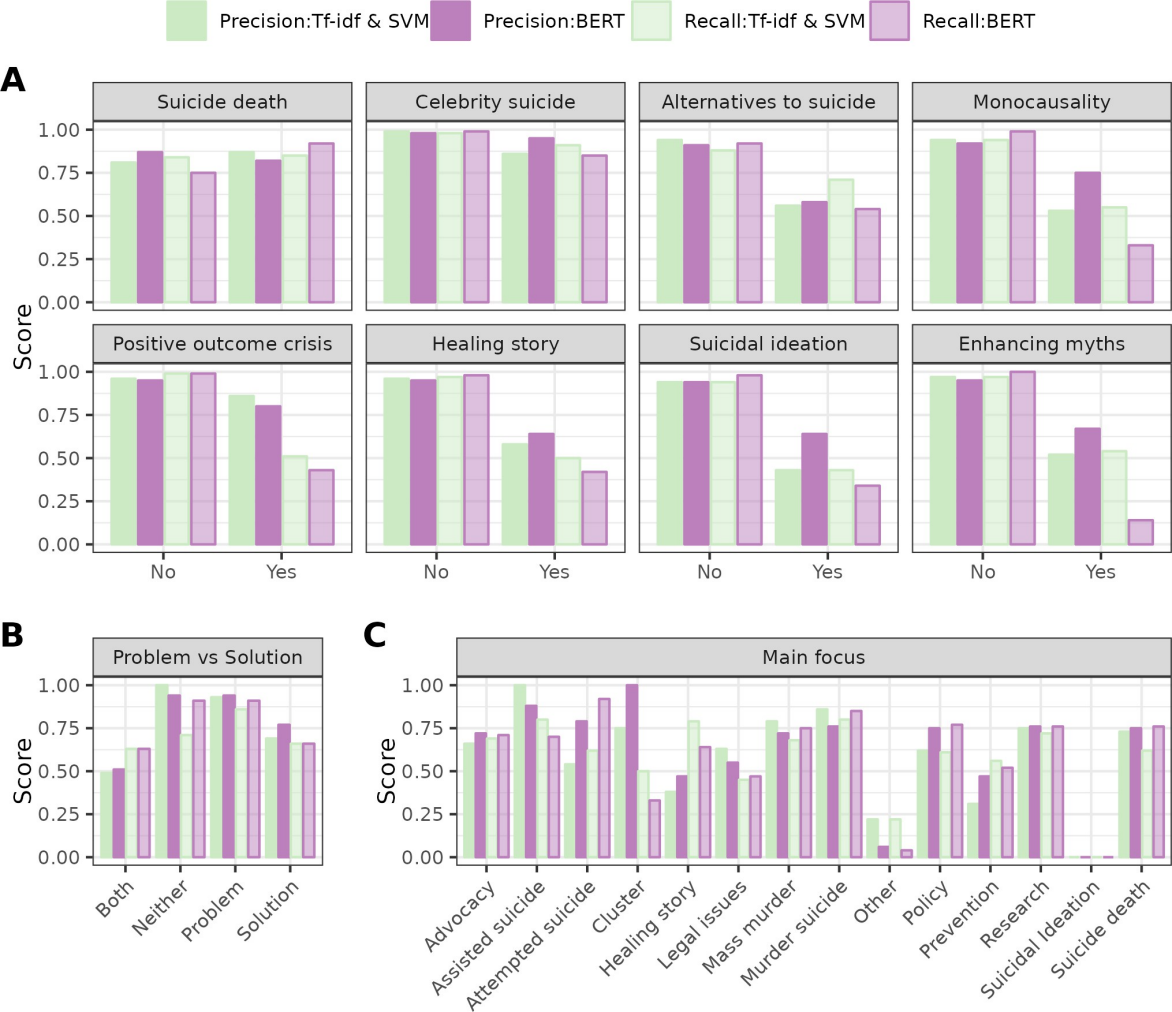
Intra-class precision and recall for Tf-idf & SVM and BERT in each classification task in the test set. (A) Binary characteristics. (B) Problem vs. solution narrative. (C) Main focus of a transcript.

We report intraclass performance scores for the two better models (BERT and Tf-idf & SVM) in the test set in S4 Table, and illustrate recall and precision for all tasks in Fig 3. Which of the two models was better varies across classification tasks, with mostly minor differences between the two models.

For most binary classification tasks (Fig 3A), both BERT and Tf-idf with SVM achieved very high performance scores (above .90) when predicting that a transcript did not contain a characteristic (precision for negative class) or detecting texts without the characteristic (recall for negative class). Predicting the less frequent presence of a characteristic (the positive class) was usually more difficult for both models (often between .50 and .60). Transcripts without the characteristic were the majority class by a large margin, except for suicide deaths, where texts mentioning a suicide were more frequent (see Fig 1A). Suicide death was also one of the two classification tasks where performance was similar for both classes (negative class: >.75, positive class: >.82). The only other such binary tasks is celebrity suicide, where models can achieve high performance by learning only the names of celebrities who died by suicide from April 1 2019 to March 30 2020, and thus do not require as many training examples.

**Fig 4:**
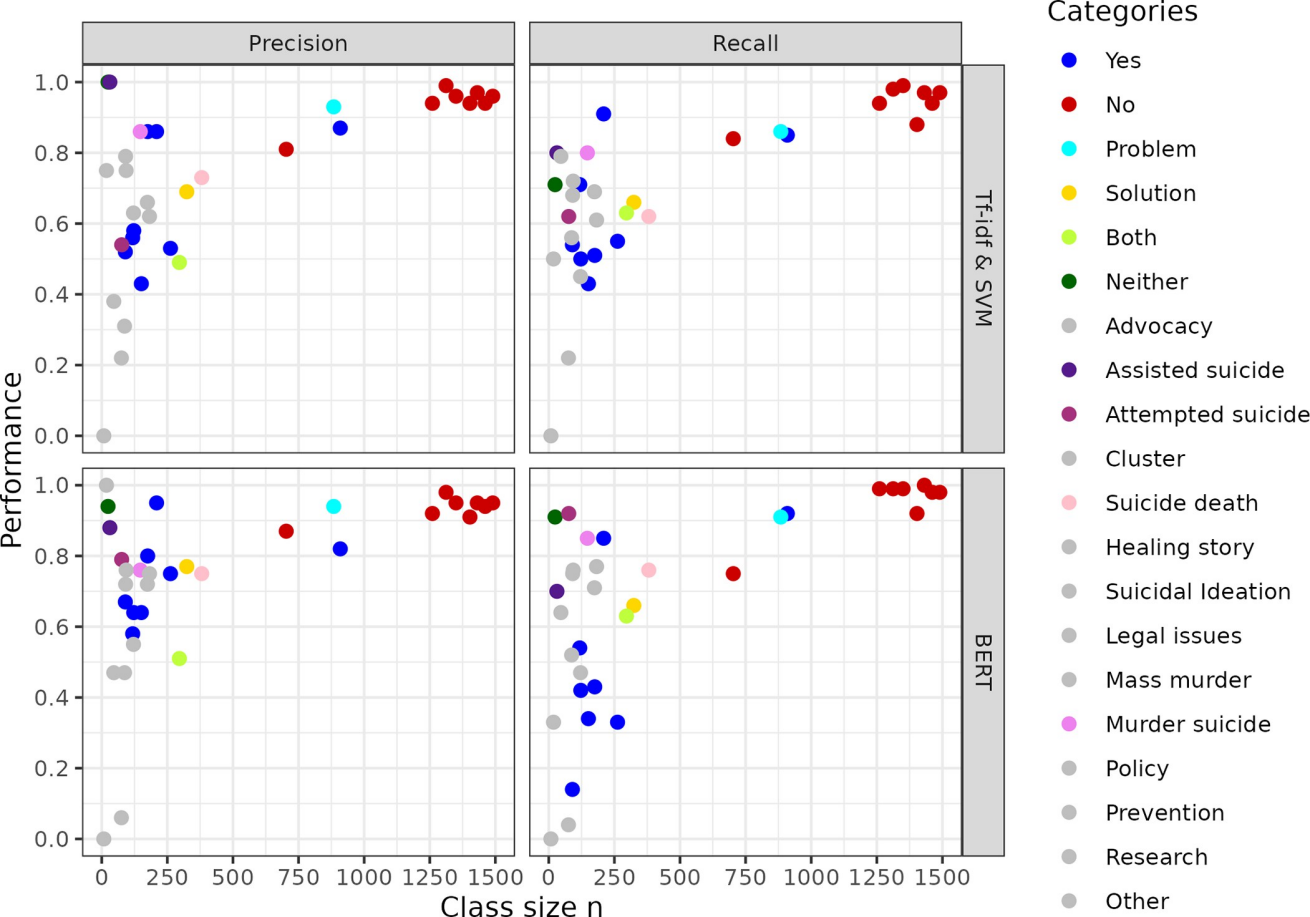
Scatter-plot of intraclass performance scores for each model with class size in the training dataset. For binary tasks, the positive (blue) and negative (red) classes have different colors. For main focus, all but three categories discussed in the main text are shown in the same color. All values displayed in this figure can be found in S4 Table.

The pattern of high performance for the negative majority class, and low performance for the rare positive class, clearly indicates that the number of texts the models could learn from had a large influence on performance scores. Indeed, Fig 4 shows strong correlations between sample size per class and precision as well as recall for both models. For class sizes above 250, performance increases steeply and linearly; for lower class sizes, performance and class size are not clearly related. The scatter plots thus reveal that sample size can explain many of the differences in classification performances between classes, in particular for class sizes above 250. Performance and sample size for the negative category is lower for suicide death (the lonely red dot outside the red at n = 703) than all other binary tasks. For positive categories, performance scores for suicide death (blue dot at n = 909) is higher than for almost all positive categories in binary tasks. Only the positive class celebrity suicide (blue dots at performance around .85-.95) can be classified even better despite lower sample size (n = 209 in training set), likely thanks to celebrity names as highly indicative keywords.

Fig 4 also reveals that sample size determines performance of multi-class tasks: Problem focus of broadcast items (cyan dot) has very similar sample sizes and performance scores as transcripts mentioning suicide deaths, although this task had four rather than two classes. Solution-focus performance (yellow dot) is much lower, as is the sample size of this class. Regarding class sizes below 250, it is interesting to note outliers with very high performance despite very low sample size: The upper left corner of some of the four panels in Fig 4 features some of the violet dots, indicating the main focus categories Assisted suicide, Murder suicide, Attempted suicide. All of these may be explained by highly indicative keywords, like “attempt”, “euthanasia” or “murder”, and”homicide”. Finally, outliers in the left bottom corner of panels, include the main focus categories Suicidal ideation and Other. With a class size of n = 2 in the test set for the first, and n = 19 for the second, these scores cannot be interpreted. The same applies for the category “Neither [problem or solution focus]” (dark green dot) with a precision of almost 1, as it had only n = 7 in the test set.

Pearson’s correlation coefficients between class size and performance scores are shown in Table 1. Correlations are reported across all categories, and for the subset of categories with a minimal number of 50 transcripts in order to exclude unreliable outlier performance scores which are based on very few labeled examples. This threshold of 50 in the training set corresponds to 15 in the test set. Applying it excludes the categories problem vs. solution focus = neither, and main focus = assisted suicide, cluster, healing story and suicidal ideation. Removing these unreliable performance scores results in correlations around .7–0.8 for both models and metrics.

**Table 1:** Correlation of class sample size in the training set with classification performance.

Model	Included categories	Precision		Recall	
		r	95%CI	r	95%CI
**Tf-idf & SVM**	**All categories**	.60	[.33, .78]	.70	[.47, .84]
	**Categories n>50**	.75	[.53, .87]	.80	[.61, .90]
**BERT**	**Categories**	.53	[.23, .73]	.65	[.39, .81]
	**Categories n>50**	.65	[.39, .83]	.69	[.43, .84]

Exceptions from the tight sample-size performance correlation may indicate which aspects of a characteristic or model additionally influence performance. A first set of exceptions are tasks which can likely be solved with a few indicative keywords, including celebrity suicides (attempted, assisted and murder suicide). Possibly, the high precision (>.80) of both models for the positive outcome of a suicidal crisis, despite the small number of positive cases to train on (n = 118), could also be explained by indicative keywords accurately identifying this characteristic.

Further, an interesting pattern across positive classes in binary tasks (except suicide death) is the generally lower recall of BERT (around .40), but often higher precision. For example, Tf-idf with SVM had a recall advantage over BERT was .22 for monocausality, .17 for alternatives to suicide, and even .40 for enhancing myths (.40). This suggests that the recall metric of BERT was especially sensitive to small class sizes, while precision did not suffer as much. BERT’s precision scores were most clearly better (by .15-.20) than Tf-idfs with SVM for moncausality, suicidal ideation and enhancing myths. Possibly, these are tasks where connotation of words, their context, and syntax matters more than for other tasks.

Finally, classifying the main focus of the item was the task with the most classes and the most severe class imbalances. Many categories had too few example transcripts to allow interpretation of their performance scores. Of more frequent categories, transcripts focusing on murder suicides were classified quite well (BERT F_1_ = .8, Tf-idf with SVM F_1_ = .83), with Tf-idf with SVM being better for precision, possibly because a few keywords (e.g., “homicide”) were sufficient to label these correctly. Reporting focusing on suicide death was classified similarly well (BERT F_1_ = .76, Tf-idf with SVM F_1_ = .67), with BERT being better at detecting many cases. This result demonstrates that at least well-defined main foci of a text, with sufficient training examples, can be classified reliably. For broader main focus topics like Policy, performance was very similar, but only with BERT (F_1_ = .76, Tf-idf with SVM F_1_ = .62). For such more complex topics, the deep learning model might provide an advantage. Finally, a main focus on Advocacy was also classified moderately well (BERT F_1_ = .72, Tf-idf with SVM F_1_ = 0.67), with similar precision and recall. In general, class imbalances as seen here are a common issue in suicide prevention, as texts featuring each characteristic are much rarer than texts without the charateristic. We tested the effect of data augmentation techniques for some of the classification tasks.^33^ The augmentation techniques did not consistently improve or otherwise change the performance scores on any of the classification tasks, suggesting that artificially augmenting rare data categories did not improve the performance of our models.^33^

## Discussion

The current study evaluated automatic labeling methods to categorize specific suicide-related content in broadcast media (i.e. radio and TV), as well as online media with machine learning. Based on a comprehensive annotation scheme for suicide-related content, we selected some content characteristics, aiming to include various classification types and difficulty levels that are relevant based on media guidelines for the reporting of suicide [3]. We then tested the ability of different machine learning models to accurately classify these characteristics, which is relevant for both research in the area of media effects as well as for surveillance efforts of suicide-related broadcast media content.

Overall, our results demonstrate that machine learning models can in principle achieve very satisfactory results for classifying various types of characteristics in suicide-related broadcast media content, including characteristics we estimated to be simpler and more difficult, as well as multi-class characteristics. This adds to earlier work that demonstrated the efficiency of machine learning in classifying social media content [11] and relevance for prevention [26]. In the current study, both the word frequency based representation Tf-idf with a linear SVM classifier as well as the deep learning model BERT were clearly better than the naive majority classifier across all tasks. However, neither BERT nor Tf-idf with SVM were clearly better than the other model. Both models also achieved similar performance in the test and validation set for almost all tasks, suggesting they generalized well to new data not seen during training.

For certain characteristics, our classifiers achieved high classification performances. The primary factor explaining these high performances, as well as the difference in performance across classification tasks, seemed to be the number of labeled examples for models to train on. Instead, if a classification required recognizing the narrative or the emotional connotation and meaning of a broadcast item, or the mere detection vs. the extent to which a characteristic was described, seemed less important. Given the importance of sample size, sound conclusions are possible only for categories that include a sufficient number of broadcast items. The first of these are all negative classes in binary tasks (a characteristic was absent), which were predicted and detected with scores above .90. This suggests that about 1250 examples may be enough to achieve highly reliable machine learning classification for suicide-related media content. Positive classes (a characteristic is present) were usually the minority class, with below 250 training samples, and their performance scores thus low (*F*_*1*_ = .4-.6) Nevertheless, the high performances for predicting the absence of these same binary characteristics suggest that machine learning models can in principle achieve very satisfactory results. The only required prerequisite seems to be more training examples for broadcast items that mention a characteristic, that is, the positive class in each task.

A second characteristic that could be classified with high precision and recall was suicide deaths reports, where sufficient examples for the negative and positive class, allowed for scores above .80. This is likely much better than what keyword searches for suicide reports can achieve, which have the problem of mixing heterogeneous types of content containing the same keywords, together. Niederkrotenthaler et al., 2020 noted accordingly that most of the research about associations of suicide reporting and subsequent suicides was based on keyword searches only, mixing together entirely different types of narratives and contents and ignoring their potentially different meaning and effects [4].

A third characteristic classified with high precision and recall (>.90) was the problem focus of a broadcast item. This is particularly encouraging, since problem focus was one out of four classes, and requires detecting what a text focuses on, rather than just whether a characteristic is present. This suggests that many characteristics of similar complexity can be recognized with machine learning tools, as long as sufficiently large training samples can be put together. Good classification performance for suicide death as one out of 14 possible main foci of broadcast items (BERT *F*_*1*_ = .76) further underlines this encouraging finding. It demonstrates that even 14 class classification problems can be solved reasonably well with as little as 400 training examples. A fifth case, solution focus, is a similar case: with only around 300 training samples, precision was around .70 in this four-class classification problem. In sum, the good performance for several complex characteristics, that require predicting which of many possible areas is the focus of a text, suggest that training models on larger samples is a very promising avenue for future research.

Regarding the featuring of a “healing story”, which have been at the forefront of the discourse of positive media potentials, ie the Papageno effect [6–9], the sample size in the current sample (there were n = 190 total healing story transcripts included) was comparable to a previous study conducted with Tweets [11]. For tweets, precision and recall using tf-idf with SVM were 0.44 (precision) and 0.64 (recall) in a 6-category classification task. For BERT, precision was 0.76 and recall was again 0.76 [11]. In the current study, sample size really determined performance (category yes with precision and recall of ~0.5 vs. no 0.97). The Twitter study suggests that having more balanced classes can improve performance for BERT for such small sample sizes.

For other classification tasks, e.g. suicidal ideation, comparisons with other samples are much more difficult, particularly because of the nature of the text analyzed. Several studies have investigated suicidal online communication and have applied machine learning particularly in order to differentiate suicidal from non-suicidal users [29–31]. Many of these models performed better as compared to the present model, which is likely due to their considerably larger sample sizes positive for suicidal ideation as compared to the present analysis.

A few points are interesting to mention regarding a comparison of Tf-idf with SVM and BERT results. Overall, we observed no clear advantage of BERT over Tf-idf with SVM. In contrast, BERT clearly outperformed Tf-idf with SVM when predicting similar suicide-related characteristics in tweets, that is, very short texts [11]. Possibly, the deep learning model’s capacity to predict the meaning of each word from the specific context it occurs in may be less crucial when working with longer content types, like the current broadcast media items. Here, we generally observed lower recall scores for BERT than Tf-idf with SVM for the positive class in all binary tasks where there were few training examples for the positive class (all except suicide death). This shows that the frequency of keywords is a useful feature to detect more instances when training sample sizes are small, and that especially the recall (the detection rate) of the deep learning model is sensitive to small class size (as here for positive classes).

Yet, at the same time, BERT often still achieved higher precision than Tf-idf with SVM for these rare positive classes. Compared to Tf-idf with SVM, BERT’s precision was most clearly better (by .15-.20) for moncausality, suicidal ideation and enhancing myths. This may hint that connotation of words, their context, and syntax matters more for these than other tasks, possibly because such descriptions are more subjective and nuanced, and simple word frequencies cannot capture this as well. Finally, BERT seemed to generalize better in the multi-class task of detecting the main focus of a text. Possibly, the ability of deep learning models to rely less on specific features of datasets helped BERT to generalize better for complex and multi-class classification tasks like detecting the main focus of a text. Taken together, the potential advantages of deep learning over word-frequency based models could not fully play out given the size of positive classes in our training data set. Still, they were recognizable in generalization abilities when training on few samples for multiple classes, and in higher precision for rarer characteristics that may require detecting nuance and changes in the meaning of words in particular contexts.

Independent from sample size, some types of classifications were indeed easier than others as we had assumed. Celebrity suicide could be classified very well (*F*_*1*_>.90) despite low positive case examples. The main focus categories assisted suicide, attempted suicide or murder-suicide are further such examples, very reasonably high performance (*F*_*1*_>.80) was possible with very low training sample sizes. However, at least in some of these cases, machine learning does not provide a substantial advantage over keyword searches, since they are easily identified with a few highly indicative keywords (names of celebrities + suicide, euthanasia, suicide bombing, attempt + suicide etc).

From a suicide prevention standpoint, the current machine learning approach developed in this work is particularly relevant when it comes to the assessment and categorization of large quantities of media items that would normally be retrieved with keyword searches only. As noted previously, searches based solely on keywords have the considerable disadvantage of mixing together entirely different narratives such as media items about celebrity suicide and suicide prevention items or items about hope and recovery [4]. The evaluation metrics of the present work clearly show that the present approach is superior to that type of keyword searching. In particular, the current findings appear helpful to make differentiations regarding characteristics that have been sufficiently represented in the current media items. This includes the determination A) if the item is about suicide reporting or not; B) includes information on celebrity suicide vs. not; C) the absence of any of the binary characteristics that were assessed (but not necessarily their presence), D) if the focus is placed on the problem or prevention of suicide; and E) if the main focus is about suicide death.

Although these categorizations do not capture all of the characteristics listed in media guidelines, they are of high relevance for suicide research and prevention as they allow for a more fine-grained categorization of texts as compared to simple keyword searches.

### Limitations and future work

Although our dataset contains a comprehensive number of media items, the size of the training dataset for specific characteristics remains a limitation of our study, as it crucially determines the performance of machine learning, in particular deep learning model [27, 28]. Especially potentially protective characteristics are not often mentioned in the media, which makes achieving high sample sizes for these characteristics challenging, but also the most promising avenue for future research and prevention efforts. This will require collecting broadcast items over longer time horizons or larger regions than our study, which included a time period of 12 months in two US states. Alternatively, different existing datasets could be merged. Care should be taken to not generalize from our results to data from other regions and time periods, without first evaluating the performance of our machine learning models there. Furthermore, we have only assessed a small number of media characteristics, far below the 39 characteristics listed in current media recommendations for suicide reporting [3]. Yet, we included several classification types, including both more information related and more subjective ones. Given that performance seemed mostly related to class size rather than the type of characteristic, it seems plausible that machine learning methods based on sufficiently large samples are effective tools for automatically labeling suicide-related content. Beyond the methods used in this paper, there are other standard machine learning approaches (e.g., naive bayesian classifier) that we have not used in the present study. The approaches selected were based on a master’s thesis conducted by one of the authors (HB) [32]. In the thesis, regarding traditional natural language processing, the naïve classifier as a trivial model and Bag.of-Words were used in addition to Tf-idf with SVM. Both of these approaches, however, were far too simplistic to capture the complexity of the present tasks.

## Conclusions

The current work makes a relevant contribution to suicide prevention research in that it investigates and confirms, for the first time, that machine learning can be successfully applied to assess prevention-relevant characteristics in broadcast media items. This included not only relative straight-forward but also more difficult characteristics of suicide-related media content such as the solution or problem focus of a media item. Recent studies about media content characteristics and their association with suicides have been limited in terms of number of media items included due to the huge amount of resources needed to assess media items. Similar limitations apply to the screening and surveillance of suicide-related media content that would allow a more pro-active approach to prevention such as the early reaching out to journalists and media professionals in instances of harmful reporting. Although this work does not replace human coding, it provides a strong basis for the extension to other media characteristics of interest, and subsequently, for the automatic assessment of large numbers of media items and their possible associations with behavioral outcomes of interest. Taken together, the current results highlight the relevance of machine learning approaches for future media studies related to suicide and prevention.

## Supporting information

S1 Table Sources of materials. (PDF)

S2 Table Coding scheme. (PDF)

S3 Table Grid search results Tf-idf & SVM. Optimal parameters indicated by grid search for TF-IDF and SVM. For SVM, balanced class weights, linear kernel, and one-vs-one decision function shape were best in all classification tasks.
(PDF)

S4 Table Class sample sizes and proportion within each task, and intraclass performance scores for all characteristics. (PDF)

S5 Table Macro-averages of performance scores (precision, recall, F1) and average accuracy in the validation and test set. (PDF)

S1 Fig Comparison of performances in validation and test set for Tf-idf with SVM and BERT. (PDF)

## References

[1] World Health Organization. Suicide data. Published 2021. Accessed February 17, 2023. https://www.who.int/teams/mental-health-and-substance-use/data-research/suicide-data

[2] World Health Organization (WHO). (2014). Preventing suicide: A global imperative. Geneva, Switzerland: World Health Organization. http://www.who.int/mental_health/suicide-prevention/world_report_2014/en/.

[3] World Health Organization. Preventing suicide: A resource for media professionals: Geneva: World Health Organization, 2017. https://apps.who.int/iris/bitstream/handle/10665/258814/WHO-MSD-MER-17.5-eng.pdf

[4] Niederkrotenthaler T, Braun M, Pirkis J, Till B, Stack S, Sinyor M et al. Association between suicide reporting in the media and suicide: systematic review and meta-analysis. BMJ. 2020;368:m575. doi: 10.1136/bmj.m575 32188637 PMC7190013

[5] Phillips DP. The influence of suggestion on suicide: substantive and theoretical implications of the Werther effect. Am Sociol Rev. 1974;39(3):340–354. 11630757

[6] Niederkrotenthaler T, Voracek M, Herberth A, Till B, Strauss M, Etzersdorfer E et al. Role of media reports in completed and prevented suicide: Werther v. Papageno effects. The British Journal of Psychiatry. 2010;197(3):234–243. doi: 10.1192/bjp.bp.109.074633 20807970

[7] Niederkrotenthaler T, Till B. Effects of suicide awareness materials on individuals with recent suicidal ideation or attempt: online randomised controlled trial. Br J Psychiatry. Published online December 17, 2019:1–8. doi: 10.1192/bjp.2019.259 31843026

[8] Niederkrotenthaler T, Till B, Kirchner S, Sinyor M, Braun M, Pirkis J et al. Effects of media stories of hope and recovery on suicidal ideation and help-seeking attitudes and intentions: Systematic review and individual participant data meta-analysis of randomised controlled trials. Lancet Public Health Volume 7, Issue 2, February 2022, Pages e156–e168. 35122759 10.1016/S2468-2667(21)00274-7

[9] Niederkrotenthaler T, Tran U, Gould M, Sinyor M, Sumner S, Strauss MJ et al. Association of Logic’s Hip Hop Song 1-800-273-8255 with Lifeline Calls and Suicides in the United States: Interrupted Time-Series Analysis. BMJ 2021;375:e067726. doi: 10.1136/bmj-2021-067726 34903528 PMC8667739

[10] Fahey RA, Matsubayashi T, Ueda M. Tracking the Werther Effect on social media: Emotional responses to prominent suicide deaths on twitter and subsequent increases in suicide. Soc Sci Med. 2018;219:19–29. doi: 10.1016/j.socscimed.2018.10.004 30342383

[11] Metzler H, Baginski H, Niederkrotenthaler T, Garcia D. Detecting Potentially Harmful and Protective Suicide-related Content on Twitter: A Machine Learning Approach. JMIR 2021: 2112.04796 [Cs]. http://arxiv.org/abs/2112.04796 10.2196/34705 PMC9434391 35976193

[12] Ophir Y, Tikochinski R, Asterhan CSC, Sisso I, Reichart R. Deep neural networks detect suicide risk from textual facebook posts. Sci Rep. 2020;10(1):16685. doi: 10.1038/s41598-020-73917-0 33028921 PMC7542168

[13] Sinyor M, Schaffer A, Nishikawa Y, Redelmeier DA, Niederkrotenthaler T, Sareen J et al. The association between suicide deaths and putatively harmful and protective factors in media reports. CMAJ. 2018;190(30):E900–E907. doi: 10.1503/cmaj.170698 30061324 PMC6066401

[14] Niederkrotenthaler T, Laido Z, Gould M, Lake AM, Sinyor M, Kirchner S et al. Associations of suicide-related media reporting characteristics with help-seeking and suicide in Oregon and Washington. Australian & New Zealand Journal of Psychiatry. 2022;0(0). doi: 10.1177/00048674221146474 36579678

[15] Pirkis JE, Burgess PM, Francis C, Blood RW, Jolley DJ. The relationship between media reporting of suicide and actual suicide in Australia. Social Science & Medicine. 2006;62(11):2874–2886. doi: 10.1016/j.socscimed.2005.11.033 16387400

[16] Hawley LL, Niederkrotenthaler T, Zaheer R, Schaffer A, Redelmeier DA, Levitt AJ et al. Is the narrative the message? The relationship between suicide-related narratives in media reports and subsequent suicides. Aust N Z J Psychiatry. 2022 Aug 23:48674221117072. doi: 10.1177/00048674221117072 Epub ahead of print. . 35999688 PMC10126449

[17] Till B, Arendt F, Rothauer P, Niederkrotenthaler T. The Role of the Narrative in Educative Suicide Awareness Materials: A Randomized Controlled Trial. Health Commun. 2023 Jan 19:1–14. doi: 10.1080/10410236.2023.2167580 Epub ahead of print. . 36659822

[18] Maiya AS. ktrain: A Low-Code Library for Augmented Machine Learning. arXiv preprint arXiv:200410703. Published online 2020.

[19] Abadi M, Agarwal A, Barham P, Brevdo E, Chen Z, Citro C et al. TensorFlow: Large-Scale Machine Learning on Heterogeneous Systems.; 2015. https://www.tensorflow.org/

[20] Pedregosa F, Varoquaux G, Gramfort A, Michel V, Thirion B, Grisel O et al. Scikit-learn: Machine Learning in Python. Journal of Machine Learning Research. 2011;12:2825–2830.

[21] Ma G. Tweets Classification with BERT in the Field of Disaster Management. Department of Civil Engineering, Stanford University; 2019.

[22] Aizawa A. An information-theoretic perspective of tf–idf measures. Information Processing & Management. 2003;39(1):45–65. doi: 10.1016/S0306-4573(02)00021-3

[23] Devlin J, Chang MW, Lee K, Toutanova K. BERT: Pre-training of Deep Bidirectional Transformers for Language Understanding. arXiv:181004805 [cs]. Published online May 24, 2019. Accessed July 14, 2021. http://arxiv.org/abs/1810.04805

[24] Liu Y, Ott M, Goyal N, Du J, Joshi M, Chen D et al. RoBERTa: A Robustly Optimized BERT Pretraining Approach. arXiv:1907.11692.

[25] Howard J, Ruder S. Universal Language Model Fine-tuning for Text Classification. arXiv:1801.06146.

[26] Niederkrotenthaler T, Tran US, Baginski H, Sinyor M, Strauss MJ, Sumner SA et al. Association of 7 million+ tweets featuring suicide-related content with daily calls to the Suicide Prevention Lifeline and with suicides, USA 2016–2018. Aust N Z J Psychiatry. 2022 Oct 14;48674221126649. doi: 10.1177/00048674221126649 36239594 PMC10947496

[27] Matykiewicz P, Pestian J. Effect of small sample size on text categorization with support vector Machines. Proceedings of the 2012 Workshop on Biomedical Natural Language Processing (BioNLP 2012), pages 193–201, Montreal, Canada, June 8, 2012.

[28] Poddar K, Amali DGB, Umadevi KS, "Comparison of Various Machine Learning Models for Accurate Detection of Fake News," 2019 Innovations in Power and Advanced Computing Technologies (i-PACT), Vellore, India, 2019, pp. 1–5, doi: 10.1109/i-PACT44901.2019.8960044

[29] Burnap P, Colombo G, Amery R, Hodorog A, Scourfield J. Multi-class machine classification of suicide-related communication on Twitter. Online Social Networks and Media. 2017;2:32–44. doi: 10.1016/j.osnem.2017.08.001 29278258 PMC5732584

[30] Bernert RA, Hilberg AM, Melia R, Kim JP, Shah NH, Abnousi F. Artificial Intelligence and Suicide Prevention: A Systematic Review of Machine Learning Investigations. International Journal of Environmental Research and Public Health. 2020;17(16):5929. doi: 10.3390/ijerph17165929 32824149 PMC7460360

[31] Sarsam SM, Al-Samarraie H, Alzahrani AI, Alnumay W, Smith AP. A lexicon-based approach to detecting suicide-related messages on Twitter, Biomedical Signal Processing and Control, 65, 2021, 102355, 10.1016/j.bspc.2020.102355.

[32] Baginski H. Automatic Detection and classification of suicide-related content in English texts. Master’s thesis. Faculty of Informatics, Vienna University of Technology, 2021. Available at: https://repositum.tuwien.at/handle/20.500.12708/17163 Accessed on February 13, 2024.

