# Supplementary material for: A machine learning approach to detect potentially harmful and protective suicide-related content in broadcast media
Source: PLoS One. 2024 May 14;19(5):e0300917. doi: 10.1371/journal.pone.0300917 (PMC11093288; doi:10.1371/journal.pone.0300917)
Supplement: S1 Table — (PDF) [file pone.0300917.s001.pdf]

Table S1. Sources of broadcast materials

OREGON		WASHINGTON
AM 860		88.5 KNKX
FM News 101 KXL		KAPP (ABC)
Fox Sports Radio 620		KAYU (FOX)
KATU-POR (ABC)		KCPQ-SEA (FOX)
KBNZ		KEPR (CBS)
KDRV (ABC)		KFFX (FOX)
KEZI (ABC)		KGMI News/Talk 790 (Radio)
KFXO (FOX)		KHQ (NBC)
KGW-POR (NBC)		KING-SEA (NBC)
KINK Radio		KIRO-AM (Radio)
KLSR (FOX)		KIRO-SEA (CBS)
KMTR (NBC)		KNDO (NBC)
KMVU (FOX)		KOMO-AM (Radio)
KOAB (PBS)		KOMO-SEA (ABC)
KOBI		KONG-SEA
KOHD (ABC)		KPLU-FM (Radio)
KOIN-POR (CBS)		KREM (CBS)
KOPB-FM (Radio)		KUOW-FM (Radio)
KPTV-POR (FOX)		KXLY (ABC)
KTVL (CBS)		Root Sports Northwest
KTVZ (NBC)		
KUIK Radio		
KVAL (CBS)		
News Radio KEX		
