# Supplementary material for: A machine learning approach to detect potentially harmful and protective suicide-related content in broadcast media
Source: PLoS One. 2024 May 14;19(5):e0300917. doi: 10.1371/journal.pone.0300917 (PMC11093288; doi:10.1371/journal.pone.0300917)
Supplement: S2 Table — (PDF) [file pone.0300917.s002.pdf]

*Table S2. Coding scheme*

| Code | Definition | Notes | Intercoder-reliability Percentage agreement; Cohen's Kappa (n) |
|---|---|---|---|
| **General characteristics** | | | |
| **Main focus** | Out of the below options, take the option that is most applicable and specific to the story. The proportion of text; framing; severity of behaviour are all relevant to this determination. If two focus points are quite similarly represented in one story, go with the more specific one. | If the focus is on suicidal ideation or behaviour of the same person, only code the most severe behaviour. | 85.0%; 0.82 (n=40) |
| suicide death | Lethal suicidal behaviour, also if suspected / unconfirmed | | |
| attempted suicide | Non-lethal suicidal behaviour, also if suspected / unconfirmed | | |
| suicidal ideation | Suicidal thoughts | The term "suicidal ideation" does not have to be used in the story to code positive. A quote such as "starting a conversation" in the context of suicide assessment, for example, would qualify as focus – suicidal ideation. | |
| murder suicide | Story on suicide in the context of homicides. Not more than 3 victims killed, otherwise code as mass murder suicide. Also homicidal / suicidal attempts qualify. | | |
| mass murder suicide | Story on suicide in the context of homicides involving more than 3 victims. Also homicidal / suicidal attempts qualify. | | |

| | | | |
|---|---|---|---|
| assisted suicide | Story about euthanasia or legal or policy issues about euthanasia. | | |
| community crisis / suicide cluster | If an story states that many or more than usual suicides have happened A) in a specific region; B) in a specific setting (e.g., all state-wide prisons); or C) in a specific short time span (weeks); or D) a specific group (natives; police...) in a short time frame (weeks); or E) in relation to a specific Internet phenomenon (e.g. Momo challenge), this qualifies as community crisis or cluster. | | |
| policy & prevention | Basically any awareness raising or prevention activity, policy initiative, healing story, is part of this code and qualifies. Further, if the story is about a law-defining formal process, it qualifies as policy. E.g., a bill that is discussed/ debated by policy-makers, or going to be signed, is „policy“, as this applies to the formal process of making law. | E.g., a bill about suicide prevention or with outlined implications for suicide prevention that is discussed/debated by policy-makers or going to be signed qualifies as policy-related. Stories about individuals or groups advocating for policy changes are to be coded as “advocacy focus”, not as policy/prevention. | |
| advocacy | Advocacy efforts are defined as organized actions taken by groups (e.g., associations of physicians) or individuals (e.g., individuals bereaved by suicide) to increase awareness, trigger legislative changes etc. These are typically grass-root, „bottom-up“ efforts; i.e. it is not a government action. | If an individual is commenting on something in response to a media request this is not sufficient to qualify as advocacy focus, even if the statement is advocating for change.<br><br>Media stories triggered by advocacy events (e.g., stories related to World Suicide Prevention Day, National Suicide Prevention Week, etc.) qualify as advocacy. | |
| suicide research | In order to qualify as research, the story needs either to refer to a specific study, or to other sources such as mortality updates. | Statements saying that "suicide is a leading cause of death among a specific group” or similar epidemiological statements without any reference to the source do not qualify as research focus. | |
| legal issues | If the story is about specific legal conflicts, i.e. about using (rather than making) law; e.g. | If the story is about the law-defining process, code as „policy“. | |

| | | | |
|---|---|---|---|
| | someone suing, pressing charges etc., code as 'legal issues'. Overall, there are two main types of coverage that are most frequently about legal issues:<br>1) Someone is suing (or is taking part in a court trial)<br>2) The cause or reason of death is unclear, and there is an investigation about it that includes legal experts (e.g., a coroner, lawyer, judge).<br>3) Stories about individual or groups protesting against or in favour of politics/political issues and targeting certain changes in the system (e.g., healthcare law) qualify as legal issues and also need to be coded as "advocacy". | If it is about advocacy for a change in law by members of the public or specific groups of non-law-makers, code as "advocacy" and "legal issues'".<br>If the pressing of charges or suing is organized to involve a group rather than individual, consider advocacy event in addition to "legal issue".<br><br>Death investigations only qualify as „legal issues", if the story clearly states that the process has reached legal experts (as opposed to first responders, e.g., the police).<br><br>Any legal issues about euthanasia are not included in here -> these qualify for "euthanasia" | |
| healing story | This is a story of hope/recovery from suicidal ideation, attempt, or bereavement from suicide. Note that the story does not have to explicitly mention a positive outcome; rather the process of finding hope and getting better qualifies as "healing story" as<br>soon as the individuals featured focus on how they have tried to cope. The outcome cannot be negative, however. | | |
| general suicide prevention | This code should be reserved for stories with focus on prevention not covered more specifically with the other prevention-related codes (i.e., healing story, advocacy efforts, or policy-prevention programme). The story may contain some reference to suicidal behaviour or ideation, but main focus needs to be placed on suicide prevention. | | |
| Other focus | Not in any of above categories | | |

| | | | |
|---|---|---|---|
| **Problem vs. solution** | The item describes that suicidal behaviour and / or suicidal ideation is a problem, or it shows how to find solutions to the problem of suicidal behaviour and/ or suicidal ideation. This item is to code the main focus in terms of being focused on the problem of suicide or how to tackle the problem of suicide. These aspects might both be represented in an item; in order to assess which one is more emphasized or if there is a "balance" between them, check the overall quantity of text devoted to the problem and solution; but also investigate the prominence / framing of both.<br>Any "solutions" can be about primary, secondary or tertiary prevention. | E.g., a text that has about equal proportions but uses much stronger wording for the solution-aspects than the problem-aspects, should still qualify as more related to a solution.<br><br>If both, problem and solution aspects come up repeatedly, with about similar amount of text and similar overall prominence, code "both".<br>Examples:<br>Problem: "every 40 seconds, someone commits suicide" or "More than 96 in-patients died by suicide in Ontario hospitals since 2007"<br>Problem: I have had those terrible thoughts non-stop, for most of my whole life. | 76.3%, 0.65 (n=118) |
| **HARMFUL CHARACTERISTICS** | | | |
| **Suicide death** | Item includes information on lethal suicidal behaviour, also if suspected / unconfirmed is part of story but not necessarily the main focus. | | 96.7%, 0.92 (n=60) |
| **Celebrity suicide** | Story reports about a suicide or suicide attempt of a celebrity. This variable applies to anyone who was famous prior to their suicidal ideation or suicidal behaviour. It does not apply if someone becomes famous due to his or her suicidal behaviour. | Also code positive for local celebrities, if there are indicators of celebrity status in the text., e.g. someone who runs a TV show. Stories about suicidal behaviours of close relatives of celebrities, e.g. their children, if they are reported based on the celebrity status of their relative (e.g. their parent), also qualify for this code. | 100%, 1 (n=60) |
| **Suggesting monocausality of suicide** | Exactly one possible motive, cause, or trigger of suicidal behaviour is reported.<br>If there are several different factors (motives, causes or triggers) in the story that are not explicitly connected with the specific suicidal act, but mentioned in the text, these factors also qualify and thus monocausality should be coded negative. | Example: If a featured person says: "The only reason for my suicidal act was depression", but the text subsequently mentions other causes/motives/triggers, e.g. unemployment, the story does not qualify as "monocausality".<br><br>If one specific cause/trigger is mentioned and some unspecific statement about further | 95%, 0.72 (n=40) |

| | | | |
|---|---|---|---|
| | | problems that were present (e.g. “he was depressed and had other problems”), this would not qualify as “monocausal”. | |
| **Enhancing/ spreading false myths about suicide** | The story enhances a false public myth on suicidal behaviour. This includes both explicit (wrong) statements and implicit statements that might make one of the myths below appear to be correct:<br>*those who talk about suicide are less likely to attempt suicide,<br>*there are no preceding warning signs,<br>*there is nothing you can do about suicidality<br>*someone who has history of attempt will not die by suicide,<br>*talking about suicide encourages suicide,<br>*only individuals with mental illness show suicidal behavior,<br>*once a person is suicidal, he or she is always suicidal,<br>*suicide can be a blessed relief for the individual or those around him/her,<br>*suicide is the only option to cope with a difficult situation. | Examples:<br>The act of desperation happened without any warning signs<br>The act was not foreseeable. None of the 19 suicides was foreseeable.<br><br>Implicit example:<br>His colleagues thought he went home happily but then they heard the lethal shot (*this statement suggests there are no warning sings—even if there were none in this case, such statements might enhance public myths*). | 100%, 1 (n=60) |
| **POSITIVE CHARACTERISTICS** | | | |
| **Suicidal ideation** | Includes a component specifically about suicidal thoughts in the absence of suicidal behaviours | The term “suicidal ideation” does not have to be used in the story to code positive. A quote such as “starting a conversation” in the context of suicide assessment, for example, would qualify as focus – suicidal ideation. | 87.5%, 0.75 (n=40) |
| **Healing story** | This item includes a story of hope/recovery from suicidal ideation, attempt, or bereavement from suicide. Note that the story does not have to explicitly mention a positive outcome; rather the | | 93.3%, 0.82 (n=30) |

| | | | |
|---|---|---|---|
| | process of finding hope and getting better qualifies as "healing story" as<br>soon as the individuals featured focus on how they have tried to cope. The outcome cannot be negative, however. | | |
| **Alternatives to suicidal behaviour** | This might include a specific action taken by an individual instead of suicidal behaviour; a suggestion /advice to seek help; advice such as "going for a walk to calm down"; or guidance on "how to make new friends". | A reference to the lifeline or another support service at the end of the text does not qualify. The alternative needs to be a part of the narrative of the story (rather than being put at the end of the text in a standardized fashion).<br><br>If the "alternative" is very general and unspecific (i.e. without any likely practical use to someone in the state of suicidal ideation), do not code as alternative. E.g., "you can get new friends" (without any additional information on how to potentially accomplish this, would not qualify).<br><br>Only code an story as positive, if the alternative is not portrayed as being ineffective or unavailable. E.g. if an story emphasises that there are many suicides, because there is no treatment available, treatment would not qualify as an "alternative", because it is portrayed as "not available".<br><br>If the story refers to some alternative (i.e., a way how to cope with bereavement) for bereaved persons, this does not generally qualify here. The story would have to specify that certain bereaved persons are at risk for dying by suicide in order to code positive for "alternative to suicidal behaviour". | 85%, 0.70 (n=40) |

| | | | |
|---|---|---|---|
| **Positive outcome of a suicidal crisis** | The story reports on a person experiencing a suicide attempt or suicidal ideation, but mastering his/her crisis or showing or accepting life-affirming or -saving behaviour. Ending is positive. | Examples: Someone calling a suicide hotline/getting help; someone making new friends.<br>Positive outcomes after bereavement only qualify if story connects bereavement with risk of suicide. | 96.7%, 0.87 (n=60) |
