# Supplementary material for: A machine learning approach to detect potentially harmful and protective suicide-related content in broadcast media
Source: PLoS One. 2024 May 14;19(5):e0300917. doi: 10.1371/journal.pone.0300917 (PMC11093288; doi:10.1371/journal.pone.0300917)
Supplement: S3 Table — Optimal parameters indicated by grid search for TF-IDF and SVM. For SVM, balanced class weights, linear kernel, and one-vs-one decision function shape were best in all classification tasks. (PDF) [file pone.0300917.s003.pdf]

Table S3. Grid search results Tf-idf & SVM

Optimal parameters indicated by grid search for TF-IDF and SVM. For SVM, balanced class weights, linear kernel, and one-vs-one decision function shape were best in all classification tasks.

**Classification task**	**Regularization parameter**	**max top features**	**n-grams (uni, bi)**
Suicide death	0.91	25000	(1,2)
Celebrity suicide	0.61	10000	(1,1)
Alternatives to suicide	0.91	25000	(1,2)
Monocausality	0.46	25000	(1,2)
Healing Story	0.76	25000	(1,2)
Suicidal ideation	0.46	25000	(1,2)
Positive outcome crisis	0.46	50000	(1,2)
Enhancing myths	0.61	10000	(1,2)
Problem vs. solution focus	0.91	10000	(1,2)
Main focus	0.91	10000	(1,1)
