# Supplementary material for: A machine learning approach to detect potentially harmful and protective suicide-related content in broadcast media
Source: PLoS One. 2024 May 14;19(5):e0300917. doi: 10.1371/journal.pone.0300917 (PMC11093288; doi:10.1371/journal.pone.0300917)
Supplement: S4 Table — (PDF) [file pone.0300917.s004.pdf]

Table S4. Class sample sizes and proportion within each task, and intraclass performance scores for all characteristics.

Classification task	Categories	Train (n, %)	Val (n, %)	Test (n, %)	Model	Precision	Recall	F_1
**Suicide death**	**Yes**	909 (56.4)	227 (56.3)	284 (56.3)	Tf-idf & SVM	0.87	0.85	0.86
					BERT	0.82	0.92	0.87
	**No**	703	176	220	Tf-idf & SVM	0.81	0.84	0.83
					BERT	0.87	0.75	0.8
**Celebrity suicide**	**Yes**	209 (13.7)	52 (13.6)	65 (13.7)	Tf-idf & SVM	0.86	0.91	0.88
					BERT	0.95	0.85	0.89
	**No**	1312	329	411	Tf-idf & SVM	0.99	0.98	0.98
					BERT	0.98	0.99	0.98
**Alternatives to suicide**	**Yes**	262 (17.2)	65 (17.1)	82 (17.2)	Tf-idf & SVM	0.56	0.71	0.62
					BERT	0.58	0.54	0.56
	**No**	1260	316	394	Tf-idf & SVM	0.94	0.88	0.91
					BERT	0.91	0.92	0.91
**Monocausality**	**Yes**	175 (11.5)	44 (11.5)	55 (11.5)	Tf-idf & SVM	0.53	0.55	0.54
					BERT	0.75	0.33	0.46
	**No**	1350	338	422	Tf-idf & SVM	0.94	0.94	0.94
					BERT	0.92	0.99	0.95
**Positive outcome crisis**	**Yes**	118 (7.8)	30 (7.9)	37 (7.8)	Tf-idf & SVM	0.86	0.51	0.64
					BERT	0.8	0.43	0.56
	**No**	1403	351	439	Tf-idf & SVM	0.96	0.99	0.98
					BERT	0.95	0.99	0.97
**Healing story**	**Yes**	122 (7.6)	30 (7.4)	38 (7.5)	Tf-idf & SVM	0.58	0.5	0.54
					BERT	0.64	0.42	0.51
	**No**	1490	373	466	Tf-idf & SVM	0.96	0.97	0.96
					BERT	0.95	0.98	0.97
**Suicidal ideation**	**Yes**	151 (9.4)	38 (9.4)	47 (9.3)	Tf-idf & SVM	0.43	0.43	0.43
					BERT	0.64	0.34	0.44
	**No**	1461	365	457	Tf-idf & SVM	0.94	0.94	0.94
					BERT	0.94	0.98	0.96
**Enhancing myths**	**Yes**	90 (5.9)	23 (6.0)	28 (5.9)	Tf-idf & SVM	0.52	0.54	0.53
					BERT	0.67	0.14	0.24
	**No**	1431	358	448	Tf-idf & SVM	0.97	0.97	0.97
					BERT	0.95	1	0.97
**Problem vs Solution**	**Problem**	884 (57.9)	221 (57.9)	277 (57.9)	Tf-idf & SVM	0.93	0.86	0.9
					BERT	0.94	0.91	0.92
	**Solution**	324 (21.2)	81 (21.2)	101 (21.1)	Tf-idf & SVM	0.69	0.66	0.68
					BERT	0.77	0.66	0.71
	**Both**	296 (19.4)	74 (19.4)	93 (19.4)	Tf-idf & SVM	0.49	0.63	0.55
					BERT	0.51	0.63	0.56
	**Neither**	24 (1.6)	6 (1.6)	7 (1.5)	Tf-idf & SVM	1	0.71	0.83
					BERT	0.94	0.91	0.92
**Main focus**	**Advocacy**	174 (11.4)	43 (11.2)	55 (11.5)	Tf-idf & SVM	0.66	0.69	0.67
					BERT	0.72	0.71	0.72

	**Assisted suicide**	31 (2.0)	8 (2.1)	10 (2.1)	Tf-idf & SVM	1	0.8	0.89
					BERT	0.88	0.7	0.78
	**Attempted suicide**	76 (5.0)	19 (5.0)	24 (5.0)	Tf-idf & SVM	0.54	0.62	0.58
					BERT	0.79	0.92	0.85
	**Cluster**	18 (1.2)	4 (1.0)	7 (1.5)	Tf-idf & SVM	0.75	0.5	0.6
					BERT	1	0.33	0.5
	**Suicide death**	381 (24.9)	96 (25.1)	121 (25.2)	Tf-idf & SVM	0.73	0.62	0.67
					BERT	0.75	0.76	0.76
	**Healing story**	46 (3.0)	12 (3.1)	14 (2.9)	Tf-idf & SVM	0.38	0.79	0.51
					BERT	0.47	0.64	0.55
	**Suicidal Ideation**	8 (0.5)	2 (0.5)	2 (0.4)	Tf-idf & SVM	0	0	0
					BERT	0	0	0
	**Legal issues**	121 (7.9)	30 (7.8)	38 (7.9)	Tf-idf & SVM	0.63	0.45	0.52
					BERT	0.55	0.47	0.51
	**Mass murder**	91 (5.9)	23 (6.0)	28 (5.8)	Tf-idf & SVM	0.79	0.68	0.73
					BERT	0.72	0.75	0.74
	**Murder suicide**	147 (9.6)	37 (9.7)	46 (9.6)	Tf-idf & SVM	0.86	0.8	0.83
					BERT	0.76	0.85	0.8
	**Policy & prevention**	182 (11.9)	45 (11.7)	57 (11.9)	Tf-idf & SVM	0.62	0.61	0.62
					BERT	0.75	0.77	0.76
	**Prevention (general)**	87 (5.7)	22 (5.7)	27 (5.6)	Tf-idf & SVM	0.31	0.56	0.39
					BERT	0.47	0.52	0.49
	**Research**	93 (6.1)	23 (6.0)	29 (6.0)	Tf-idf & SVM	0.75	0.72	0.74
					BERT	0.76	0.76	0.76
	**Other**	75 (4.9)	19 (5.0)	22 (4.6)	Tf-idf & SVM	0.22	0.22	0.22
					BERT	0.06	0.04	0.05
