# Supplementary material for: A machine learning approach to detect potentially harmful and protective suicide-related content in broadcast media
Source: PLoS One. 2024 May 14;19(5):e0300917. doi: 10.1371/journal.pone.0300917 (PMC11093288; doi:10.1371/journal.pone.0300917)
Supplement: S5 Table — (PDF) [file pone.0300917.s005.pdf]

Table S5. Macro-averages of performance scores (precision, recall, F_1) and average accuracy in the validation and test set

Classification task	Model	Validation set			Test set		
		Prec.	***Recall***	***F1***	***Prec.***	***Recall***	***F1***
**Suicide death**	*Majority*	0.28	0.50	0.36	0.28	0.50	0.36
	Tf-idf & SVM	0.83	0.82	0.83	0.84	0.85	0.84
	BERT	0.85	0.84	0.84	0.85	0.83	0.84
**Celebrity suicide**	*Majority*	0.43	0.50	0.46	0.43	0.50	0.46
	Tf-idf & SVM	0.92	0.93	0.92	0.92	0.94	0.93
	BERT	0.99	0.96	0.98	0.96	0.92	0.94
**Alternatives to suicide**	*Majority*	0.42	0.50	0.45	0.41	0.50	0.45
	Tf-idf & SVM	0.75	0.81	0.77	0.75	0.80	0.77
	BERT	0.80	0.78	0.79	0.74	0.73	0.73
**Monocausal explanation**	*Majority*	0.44	0.50	0.47	0.44	0.50	0.47
	Tf-idf & SVM	0.71	0.68	0.69	0.73	0.73	0.74
	BERT	0.70	0.57	0.59	0.83	0.66	0.70
**Positive outcome crisis**	*Majority*	0.46	0.50	0.48	0.46	0.50	0.48
	Tf-idf & SVM	0.88	0.71	0.77	0.91	0.75	0.81
	BERT	0.92	0.75	0.81	0.88	0.71	0.77
**Healing story**	*Majority*	0.46	0.50	0.48	0.46	0.50	0.48
	Tf-idf & SVM	0.84	0.77	0.80	0.77	0.74	0.75
	BERT	0.91	0.70	0.76	0.80	0.70	0.74
**Suicidal ideation**	*Majority*	0.45	0.50	0.48	0.45	0.50	0.48
	Tf-idf & SVM	0.68	0.65	0.66	0.69	0.68	0.69
	BERT	0.78	0.65	0.69	0.79	0.66	0.70
**Enhancing myths**	*Majority*	0.47	0.50	0.49	0.47	0.50	0.46
	Tf-idf & SVM	0.72	0.72	0.72	0.74	0.75	0.75
	BERT	0.72	0.56	0.59	0.81	0.57	0.60
**Problem vs solution**	*Majority*	0.15	0.25	0.18	0.15	0.25	0.18
	Tf-idf & SVM	0.76	0.74	0.75	0.78	0.72	0.74
	BERT	0.73	0.67	0.68	0.77	0.77	0.77
**Main focus**	*Majority*	0.02	0.07	0.03	0.02	0.07	0.03
	Tf-idf & SVM	0.68	0.68	0.68	0.59	0.58	0.57
	BERT	0.59	0.60	0.59	0.62	0.59	0.59
