# Supplementary figures and images for: A machine learning approach to detect potentially harmful and protective suicide-related content in broadcast media
Source: PLoS One. 2024 May 14;19(5):e0300917. doi: 10.1371/journal.pone.0300917 (PMC11093288; doi:10.1371/journal.pone.0300917)

Figure S1. Comparison of performances in validation and test set for Tf-idf with SVM and BERT

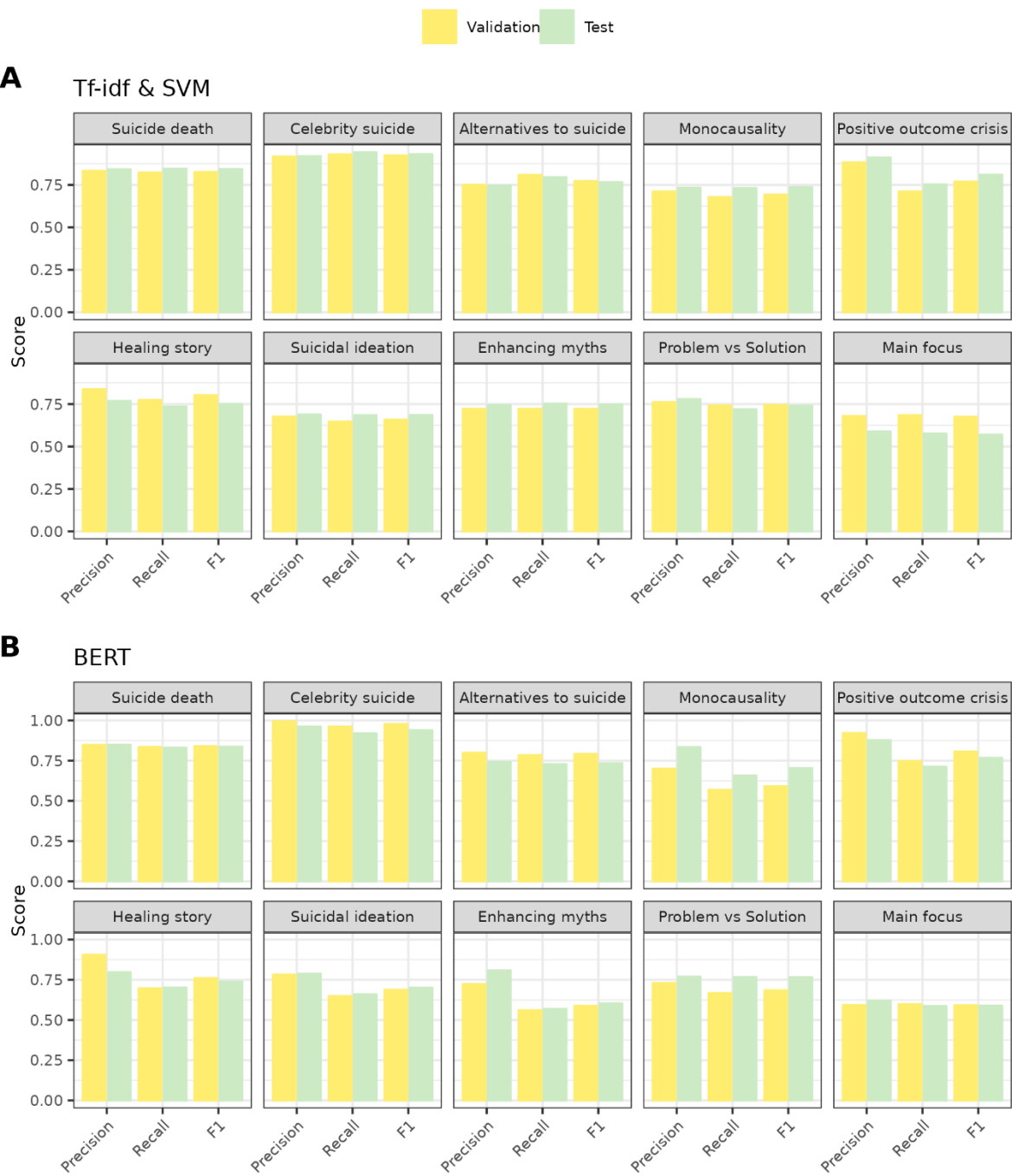

Supplement: S1 Fig — (PDF) [file pone.0300917.s006.pdf]
